# Using Raw Accelerometer Data to Predict High-Impact Mechanical Loading

**DOI:** 10.3390/s23042246

**Published:** 2023-02-16

**Authors:** Lucas Veras, Florêncio Diniz-Sousa, Giorjines Boppre, Vítor Devezas, Hugo Santos-Sousa, John Preto, João Paulo Vilas-Boas, Leandro Machado, José Oliveira, Hélder Fonseca

**Affiliations:** 1 Research Center in Physical Activity, Health and Leisure (CIAFEL), Faculty of Sport, University of Porto, 4200-450 Porto, Portugal; 2 Laboratory for Integrative and Translational Research in Population Health (ITR), University of Porto, 4200-450 Porto, Portugal; 3 Obesity Integrated Responsability Unity (CRIO), São João Academic Medical Center, 4200-319 Porto, Portugal; 4 Center of Research, Education, Innovation and Intervention in Sport (CIFI2D), Faculty of Sport, University of Porto, 4200-450 Porto, Portugal; 5 Biomechanics Laboratory (LABIOMEP-UP), University of Porto, 4200-450 Porto, Portugal

**Keywords:** ground reaction force, loading rate, jumps, validation, biomechanics

## Abstract

The purpose of this study was to develop peak ground reaction force (pGRF) and peak loading rate (pLR) prediction equations for high-impact activities in adult subjects with a broad range of body masses, from normal weight to severe obesity. A total of 78 participants (27 males; 82.4 ± 20.6 kg) completed a series of trials involving jumps of different types and heights on force plates while wearing accelerometers at the ankle, lower back, and hip. Regression equations were developed to predict pGRF and pLR from accelerometry data. Leave-one-out cross-validation was used to calculate prediction accuracy and Bland–Altman plots. Body mass was a predictor in all models, along with peak acceleration in the pGRF models and peak acceleration rate in the pLR models. The equations to predict pGRF had a coefficient of determination (R^2^) of at least 0.83, and a mean absolute percentage error (MAPE) below 14.5%, while the R^2^ for the pLR prediction equations was at least 0.87 and the highest MAPE was 24.7%. Jumping pGRF can be accurately predicted through accelerometry data, enabling the continuous assessment of mechanical loading in clinical settings. The pLR prediction equations yielded a lower accuracy when compared to the pGRF equations.

## 1. Introduction

Bones respond to mechanical loading by altering their properties, such as mass, microarchitecture, and geometry, which ultimately dictate their strength and resistance to fracture [1,2,3]. Therefore, physical activity is a paramount stimulus to maintain and improve bone health throughout the life course [4]. However, the osteogenic effect of physical activity is highly dependent on the characteristics of mechanical loading [5]. Several studies on experimental animals [1,6,7] have helped to form our understanding of the principles that drive bone tissue’s adaptive response to mechanical loading. In general, high-magnitude dynamic impact loading applied at high frequencies and including long rest periods seems to favor increases in bone mass and strength [1]. This seems to corroborate the available evidence from humans. For instance, findings from different types of sports have shown that athletes practicing sports involving high- and/or odd-impact loading (e.g., volleyball and football) have a greater bone mass and estimated bone strength compared to repetitive and low-impact loading sports (e.g., cycling and swimming) [8]. Nevertheless, the precise way in which the manipulation of mechanical loading variables influences bone tissue’s adaptive response in humans is not fully understood, particularly due to the difficulty in accurately assessing bone’s mechanical loading and its components in vivo in humans.

Some questionnaires have been developed in an attempt to capture the bone-stimulating characteristics of specific activities [9,10]. However, although the scores generated by such subjective methods seem to be related to bone status, this association appears to be limited and inconsistent across studies [11,12,13]. A more robust approach to assess mechanical loading is the use of equipment such as force plates—the gold standard for ground reaction force measurement [14]. However, the use of force plates is less applicable in some clinical and research settings, as they are immobile and expensive devices that are limited to assessments in laboratory conditions. In the last few years, objective methods based on wearable devices have been proposed as a solution to overcome these limitations, since acceleration and some mechanical loading variables resulting from body movement have shown to be correlated [15,16]. In fact, several studies using different prediction model approaches (e.g., linear regression, linear mixed models, machine learning) have already shown that, through the use of acceleration data, it is possible to estimate some mechanical loading components that are typically assessed only through force plates, namely, peak ground reaction force (pGRF) [17,18,19,20,21,22,23,24,25] and peak loading rate (pLR) [19,21,22]. Although accelerometry-based equations have shown high prediction accuracy [21,22], this versatile approach to mechanical loading assessment is not widely used yet, either in clinical practice or research. One of the issues that may contribute to this fact is the lack of validation studies covering a broader spectrum of activities, especially those known to have a greater osteogenic effect, such as high-impact activities.

Until now, studies that have calibrated accelerometers to predict mechanical loading variables have usually employed only simple activities, such as walking [19,20,21] or walking and running [17,18,22,24], while activities eliciting higher impact loading have not yet been tested. The fact that these accelerometry-based prediction equations are only valid for a limited set of activities hinders their ability to accurately quantify the amount of loading to which a person is subjected daily, as the physical activities performed daily are often composed of other types of activities than just walking and running. Therefore, the validity of these methods should be expanded to other activities that are commonly associated with positive bone adaptations. Jumping exercises, for instance, are frequently used as a strategy to improve bone health in post-menopausal women [26] and elderly men [27]. The direct measurement of the mechanical loading involved in these activities would greatly improve our ability to precisely control the loading regimen elicited by these physical exercises, and to establish a more reliable relationship between mechanical stimulation and the bone’s anabolic response and, consequently, to establish a clearer relationship between exercise dose and the extent of the bone’s adaptive response [28]. So far, accelerations recorded from body-worn devices have been shown to be correlated with ground reaction forces during jumping [15], and accelerometers have also been successfully used to predict other biomechanical variables during jumping activities [29]. Therefore, the objective of this study was to develop accelerometry-based pGRF and pLR prediction equations for high-impact activities in adult subjects with a broad range of body masses, from normal weight to severe obesity.

## 2. Methods

**Figure 1:**
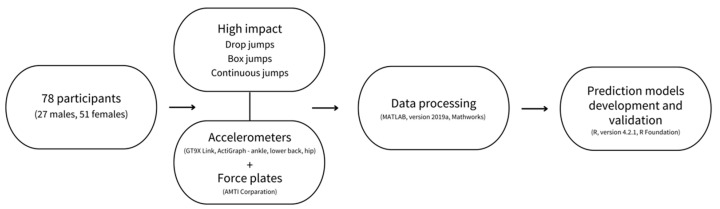
Overall scheme of the study design and methods employed in the study.

An overall scheme of the experimental design and methods employed in this study can be observed in Figure 1.

### 2.1. Participants

A total of 78 adults participated in the study (27 males: 34.0 ± 12.3 years, 173.2 ± 6.3 cm, 83.2 ± 14.9 kg, 27.9 ± 5.6 kg·m^−2^; 51 females: 36.5 ± 11.2 years, 158.1 ± 6.8 cm, 82.0 ± 23.2 kg, 33.0 ± 9.6 kg·m^−2^; $\bar{X}$ ± SD). The protocol was approved by the local ethics committee (CES 192-14), and all participants were informed about the experiments’ purpose and protocol before giving their written informed consent. The inclusion criteria were as follows: age between 18 and 65 years, and the ability to perform the jumps included in the trials. The exclusion criterion was the presence of any acute injury or chronic limitation precluding any of the experimental exercises. The measurement of height (stadiometer model 213; Seca, Hamburg, Germany) and body mass (digital scale model 899; Seca, Hamburg, Germany) was carried out according to standard procedures [30].

### 2.2. Protocol

**Figure 2:**
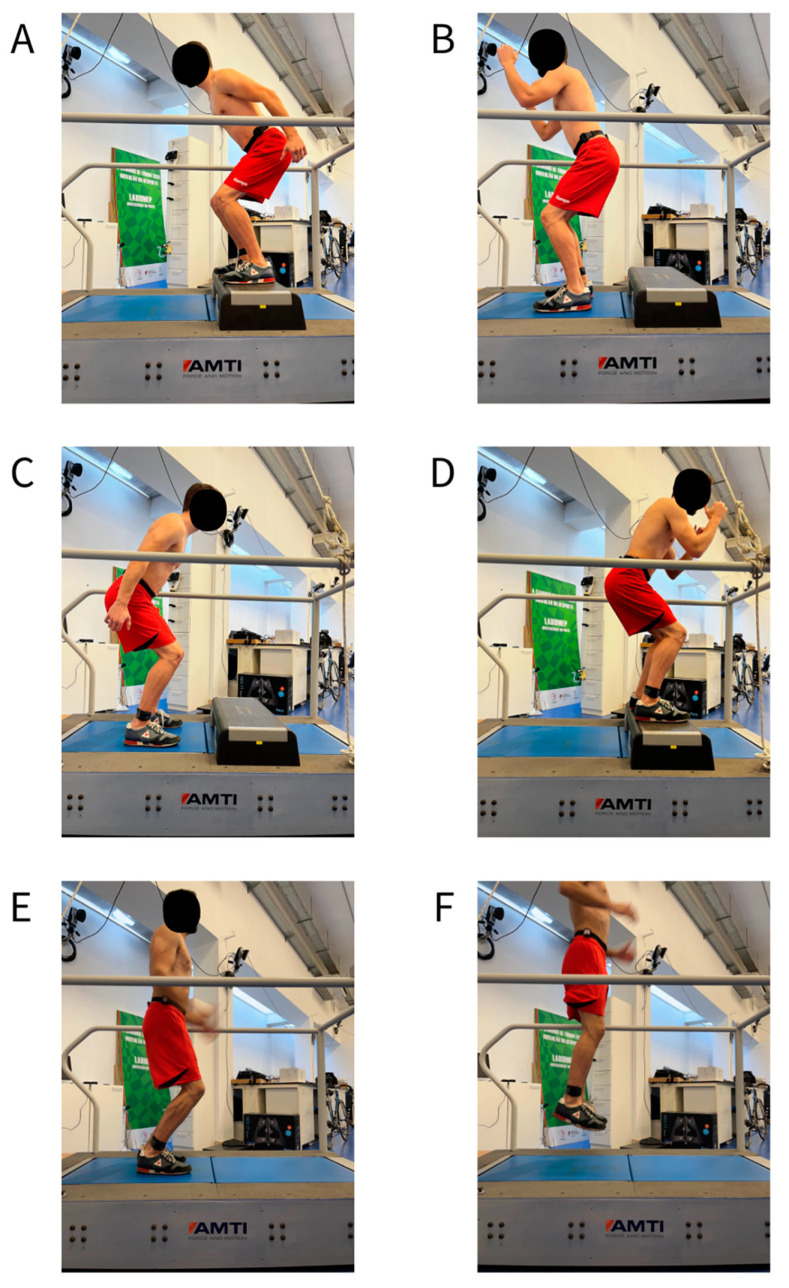
An example of a participant performing each of the jump types assayed. Panels (**A**,**B**) show the initial (take-off phase) and final (landing phase) phases of the drop jumps, respectively, while Panels (**C**,**D**) show the initial and final phases of the box jumps, respectively, and Panels (**E**,**F**) show the initial and final phases of the continuous jumps, respectively.

The participants completed a series of trials involving jumps of different types and from different heights. First, drop jumps were executed from steps varying from 5 cm to 40 cm high, with 5 cm increments. Then, box jumps were performed starting from the floor up to 5 cm, 15 cm, and 30 cm high boxes. Finally, continuous jumps were carried out at two different heights: 5 cm and 15 cm. Drop and box jumps were executed in two sets of four jumps at each height, with a 30 s interval, and the continuous jumps were performed in one set of 20 s at each height. Before starting the data collection, the participants performed a few repetitions of each jump type—always at the lowest height—to familiarize themselves with the protocol. The jumps were always performed in the following order: drop jumps, box jumps, continuous jumps—and from lower to higher heights. The landing phase of the drop jumps, the take-off phase of the box jumps, and both phases of the continuous jumps were recorded on a force plate at a 1000 Hz sampling frequency (AMTI Corporation, Watertown, USA). The participants were instructed to jump with no restrictions regarding their arm movements, to land two-footed, and to remain stationary on the force plate for around five seconds after landing (except for the continuous jumps). To better characterize the types of jumps performed, the initial and final position of each jump can be seen in Figure 2.

**Figure 3:**
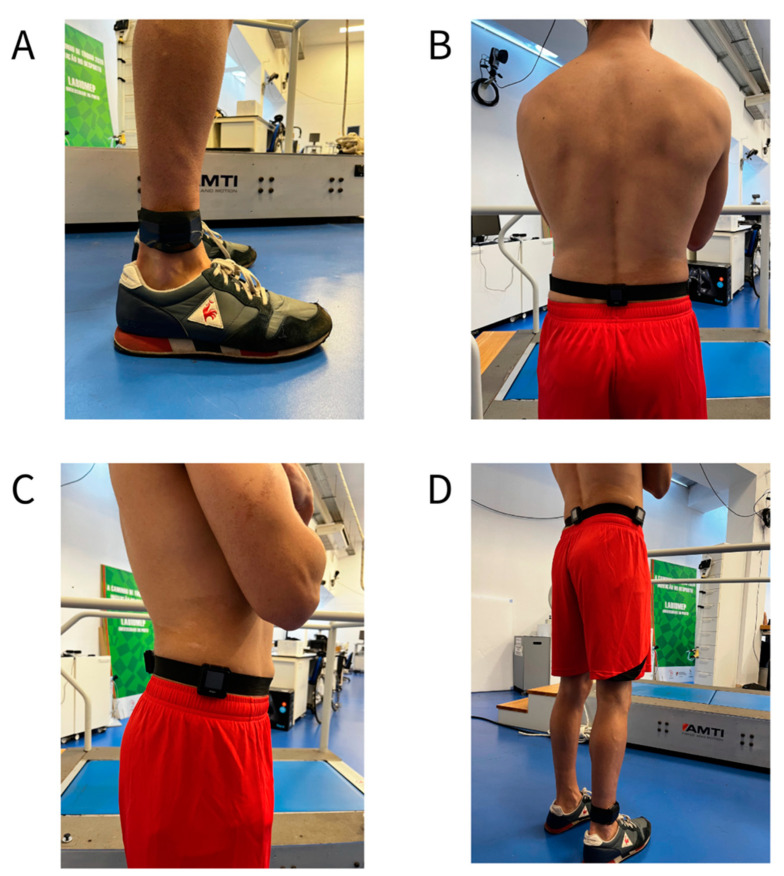
Accelerometers’ positioning on the body: Details of accelerometer positioning on the ankle, lower back, and hip are displayed in Panels (**A**–**C**), respectively. Panel (**D**) shows all accelerometer placements simultaneously.

Throughout the protocol, three wearable sensors (GT9X Link; 100 Hz; ±16 g range; ActiGraph, Pensacola, FL, USA) were worn by each participant, at the following placements: (i) right hip (along the anterior axillary line, at the level of the iliac crest); (ii) lower back (at the midpoint between the two posterior superior iliac spines); and (iii) right ankle (immediately superior to the lateral malleolus). The sensors worn at the hip and lower back were positioned on the same tightly secured elastic belt with clips, and the ankle-worn sensor was fixed by an elastic belt and adhesive tape. The wearable sensors were always positioned to ensure the alignment of the accelerometer’s vertical axis and the standing body’s longitudinal axis. The accelerometers’ positioning on the body can be seen in Figure 3. The hip and lower back placements were chosen due to their proximity to the body’s center of mass, and the ankle placement was chosen because its location is closer to the ground contact point. The wearable sensors were set to collect data at 100 Hz, which is the device’s maximum sampling frequency.

Each wearable sensor used includes a primary and a secondary triaxial accelerometer. As the manufacturer’s proprietary filter is not applied to the secondary accelerometer’s raw data, only data from this accelerometer were used in this study. This increases the accuracy and replicability of data collection and processing. Data from the force plates (Netforce, Version 3.5.1; AMTI Corporation, Watertown, USA) and accelerometers (ActiLife version 6.13.3; ActiGraph, Pensacola, USA) were operated through the manufacturer-supplied software and exported as raw data from the *x*, *y*, and *z* vectors. The ground reaction force was expressed in newtons (N), and acceleration was expressed in gravitational acceleration units (1 *g* = 9.807 m·s^−2^).

### 2.3. Data Processing

**Figure 4:**
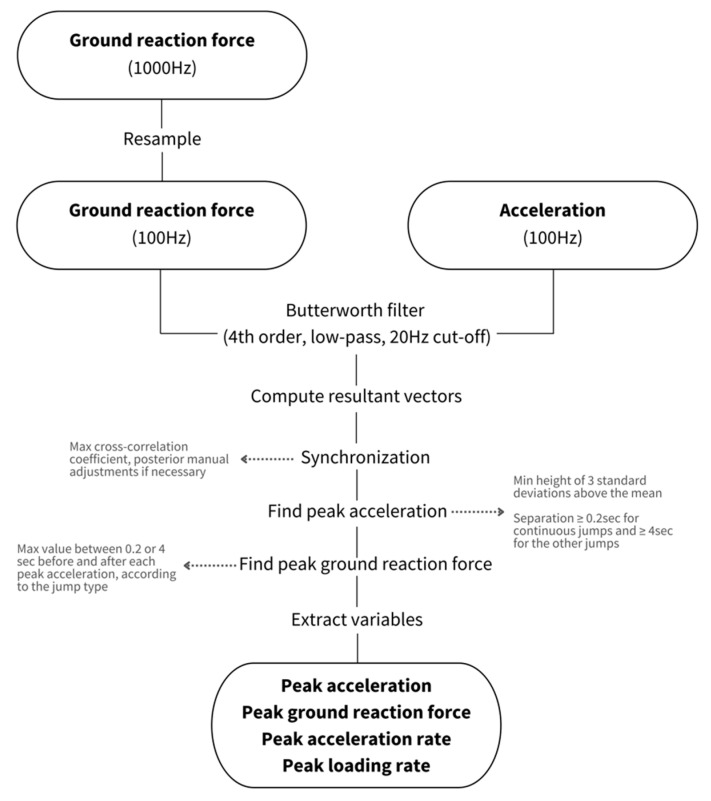
Flowchart showing all steps of the data processing.

**Figure 5:**
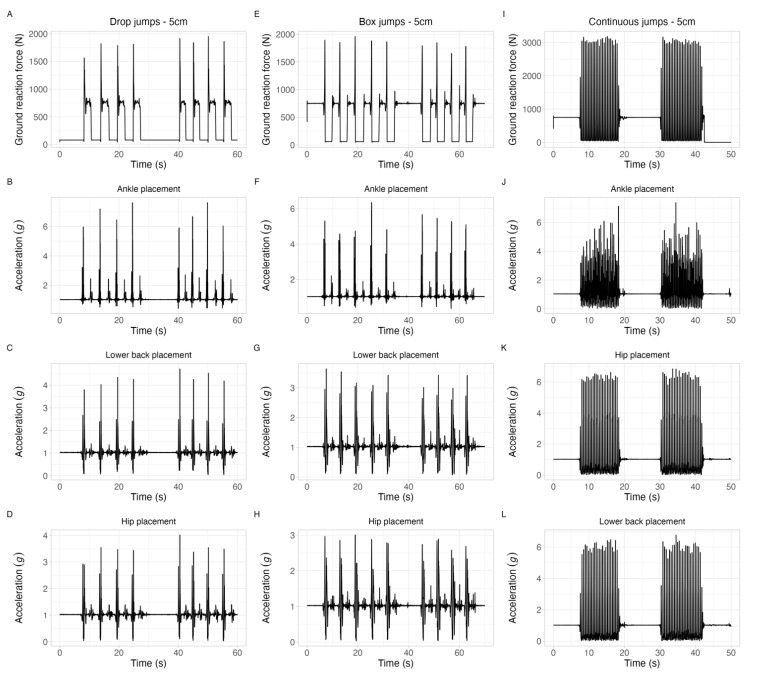
Examples of the ground reaction force and acceleration signals for all of the jump types performed. The first column, formed by Panels (**A**–**D**), shows the ground reaction force and acceleration signal for accelerometers at the ankle, lower back, and hip for the drop jump from 5 cm height. Panels (**E**–**H**) show the same signals, but for the box jumps of 5 cm height, while Panels (**I**–**L**) show the signals for the continuous jumps of 5 cm.

A flowchart showing the steps of the data processing can be seen in Figure 4. MATLAB software (Version 2019a, MathWorks, Natick, MA, USA) was used to process the data from both the force plates and the accelerometers. Data were processed according to the following procedures: First, the ground reaction force signal from the force plate (1000 Hz) was resampled to match the activity monitors’ sampling frequency (100 Hz). Then, both the ground reaction force and acceleration signals were filtered using a Butterworth fourth-order low-pass filter, with a 20 Hz cutoff frequency, to attenuate the noise. This cutoff frequency was selected based on visual inspection of the signals’ frequency spectra after a fast Fourier transform (FFT). Afterwards, the ground reaction force and acceleration’s resultant vector was calculated ($r_{i}=\sqrt{x_{i}^{2}+y_{i}^{2}+z_{i}^{2}}$). After that, both signals were adjusted using the time set by the system’s clock and then synchronized by the maximum cross-correlation coefficient. Then, the ground reaction force and acceleration signals were visually inspected, and whenever their peaks were not in the same hundredth of a second, manual adjustments to the synchronization were performed. An example of the ground reaction force and acceleration signals for each jump type can be observed in Figure 5. Next, the peak acceleration (pACC) was determined by the following criteria: (i) a minimum magnitude of three standard deviations above the mean acceleration value recorded during a trial, and (ii) a separation of at least 0.2 s for continuous jumps and 4 s for the other jumps. Then, pGRF was defined as the highest value within +/− 0.2 (continuous jumps) or +/− 4 (drop and box jumps) seconds of each pACC, according to the jump type. The rates of change were computed through a centered derivative from the beginning of the foot contact to the curve peak (Equation (1)). The pLR and peak acceleration rate (pAR) were defined as the maximum value in the array derived from Equation (1) and represented by $f_{i_{max}}^{\prime }$ in Equation (2).

(1) $$f_{i}^{\prime }=\frac{f_{i+1}-f_{i-1}}{t_{i+1}-t_{i-1}}$$

(2) $$f_{i_{max}}^{\prime }=max\left(f_{i}^{\prime }\right)\;$$

Finally, the mean pGRF, pACC, pLR, and pAR of the resultant vector and its vertical component for each participant at each jump type and height for each accelerometer placement were extracted and used in all subsequent analyses.

### 2.4. Statistical Analyses

Statistical analyses were performed using R statistical software (version 4.2.1, R Foundation for Statistical Computing, Vienna, Austria). All code used in the data analysis is registered in an open platform (https://bit.ly/3szNSJS, accessed on 31 October 2022). Statistical significance was set as α = 0.05.

Linear mixed models were used to develop pGRF and pLR prediction equations for each vector (resultant and vertical) and accelerometer placement (ankle, lower back, and hip). Body mass was tested as a predictor in all models, along with the acceleration magnitude (pACC) in the pGRF models and its rate (pAR) in the pLR models. These predictors were entered as fixed effects and kept in the model, as all were shown to be significant (*p* < 0.05). The subject and the interaction between jump type and jump height were tested as random effects, and both factors were shown to improve the models. The final models were chosen according to the −2 log-likelihood statistic [31]. The conditional coefficient of determination (R^2^), which estimates the variance explained by both fixed and random factors [32], was computed.

Model validation was performed using the leave-one-out cross-validation method [33]. This method consists of separating data from one of the subjects into a testing dataset while keeping the remainder in the training dataset. Then, a new model is developed using the training dataset, with the same parameters that were defined using the entire sample. This new model is then applied to predict the outcome for the subject in the testing dataset. The whole process is repeated once for each participant (78 times). Data from the testing dataset were used in all subsequent analyses.

Model quality was then evaluated both numerically and graphically. The numerical assessment was performed by computing the following accuracy indices: mean absolute error (MAE), mean absolute percentage error (MAPE), and root-mean-square error (RMSE). To visually analyze the agreement between the values predicted by the models and those measured by the force plates, Bland–Altman plots were drawn. These plots are constructed by plotting the mean of the actual and predicted values on the *x*-axis and their difference on the *y*-axis. Then, the bias was calculated as the mean of these differences and plotted as a continuous horizontal line. The limits of agreement were defined as bias ± 1.96 standard deviation and plotted as horizontal dashed lines [34]. To further test whether there was any systematic under- or overestimation of the values by the models, one-sample *t*-tests were performed to check whether the bias were significantly different from zero. Finally, linear regressions were employed to examine whether the difference between the actual and predicted values was affected by their magnitude, thereby verifying whether the prediction was constant throughout the entire magnitude range [35].

## 3. Results

**Figure 6:**
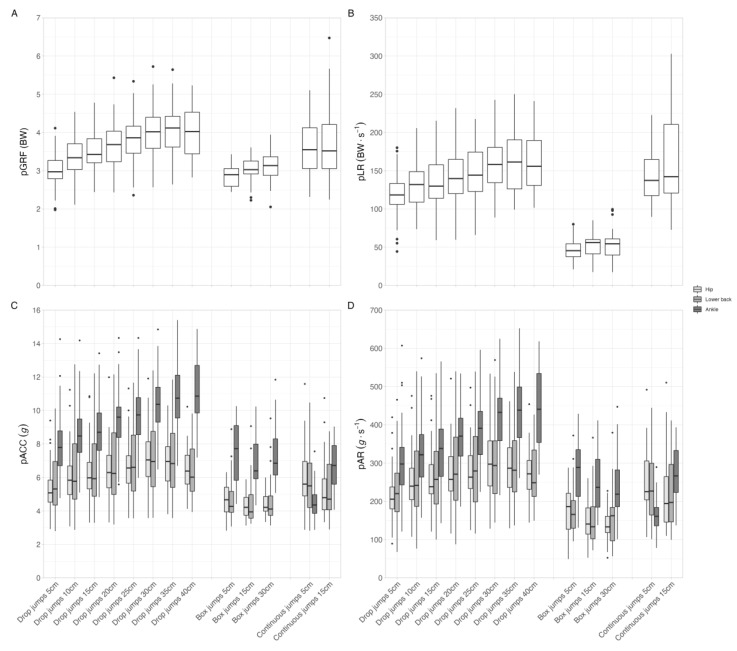
Distribution of the resultant vectors of mechanical loading (peak ground reaction force on Panel (**A**) and peak loading rate on Panel (**B**)) and acceleration (peak acceleration on Panel (**C**) and peak acceleration rate on Panel (**D**)) variables per jump type and height combination. Abbreviations: BW, body weight; pACC, peak acceleration; pAR, peak acceleration rate; pGRF, peak ground reaction force; pLR, peak loading rate.

In our protocol, three distinct jump types were performed, each using several different heights. This variety of jumping trials produced a considerable heterogeneity in the response of both the force-plate- and accelerometer-measured variables. The results obtained for pGRF, pLR, pACC, and pAR, with respect to their resultant vectors, can be observed in Figure 6 (see Supplementary Figure S1 for the vertical vector).

Panels A and B show the force-plate-measured variables normalized by the subject’s body mass, and by the pGRF and pLR, respectively. It can be seen that, in the drop jump trials, there is a tendency for the loading magnitude to increase as the jump height increases. This tendency is also observed for the box jumps, but with a lower magnitude. As for the continuous jumps, both assayed heights elicited reasonably similar loading profiles. Panels C and D show the values of the accelerometer-measured variables—pACC and pAR, respectively—for each trial. It can be observed that, for all jump types and heights, data from ankle-worn accelerometers typically record higher values compared to data from the accelerometers placed at the lower back and hip. Moreover, during the drop jumps, there is a constant increase in the values from the ankle accelerometers as the jump height increases, while values from the lower back and hip accelerometers tend to plateau from 30 cm height onwards. As for the box jumps, the pACC values remain reasonably constant throughout the assayed heights, whereas pAR tends to slightly decrease as the jump heights increase. Lastly, for the continuous jumps, both pACC and pAR from the lower-back- and hip-worn accelerometers marginally decrease when comparing the 5 cm to 15 cm jumps. However, the opposite is true for the ankle accelerometers, with their pACC and pAR increasing from 5 cm to 15 cm height.

Accelerometer-based mechanical loading prediction equations were developed based on linear mixed models. The regression coefficients, R^2^, and accuracy indices of the models derived from accelerometers worn at the ankle, lower back, and hip, and from the resultant and vertical vectors, are presented in Table 1. The R^2^ values for the pGRF models ranged from 0.83 to 0.92, showing that all of the models were able to explain at least 80% of the pGRF variance, and the observed MAPE values were between 12.3% and 14.5%. Despite the similar accuracy results achieved by all equations, the model derived from data from the resultant vector of accelerometers worn at the hip was shown to have the lowest accuracy error, represented by the smallest MAE (302.1 N), MAPE (12.3%), and RMSE (396.6 N).

Compared to the pGRF models, the pLR equations yielded poorer results. Although their R^2^ ranged from 0.87 to 0.91, their error measures were high, with MAPE ranging from 20.7% to 24.7%. Nevertheless, similar to the pGRF models developed, the best pLR prediction equation in terms of prediction accuracy was the one from the resultant vector derived from hip-worn accelerometers, even though its error estimates were higher, with an MAE of 16812 N, MAPE of 20.7%, and RMSE of 21545 N.

**Figure 7:**
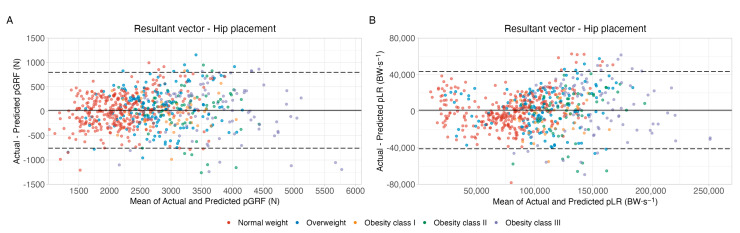
Bland–Altman plots showing agreement between actual and predicted peak ground reaction force (Panel **A**) and loading rate (Panel **B**) for the resultant vectors from hip-worn accelerometers. Continuous thick lines show bias (the average of the differences between actual and predicted values), while dashed lines show the limits of agreement (bias ± standard deviation). Abbreviations: pGRF, peak ground reaction force; pLR, peak loading rate.

To visualize the agreement between the pGRF and pLR values measured by the force plates and predicted by the models, a series of Bland–Altman plots were built for both the resultant and vertical vectors and for all accelerometer placements. Figure 7 shows the Bland–Altman plots for the resultant pGRF (panel A) and pLR (panel B) vectors of the hip-worn accelerometers. Both plots show good agreement levels, as at least 94% of the data points are within the limits of agreement and the data tend to cluster around zero. Bland–Altman plots for the other accelerometer placements and vectors also showed the same previously described patterns and can be found in Supplementary Figure S2 for pGRF and in Supplementary Figure S3 for pLR. Moreover, as could be observed from the results of the one-sample *t*-tests, none of the equations presented a bias significantly different from zero (*p* > 0.05). Furthermore, all of the equations showed a proportional bias, i.e., the magnitude of the values influences the prediction error. However, as the linear regressions used to assess the proportional bias presented a very small R^2^ (maximum value of 0.10), this effect is negligible.

Finally, an R package was also developed [36] to simplify the application of all of the necessary data processing steps involved in the implementation of the developed prediction equations. The package website (https://verasls.github.io/impactr, accessed on 31 October 2022) has all of the installation instructions and documentation necessary to apply this method on the user’s own data.

## 4. Discussion

This study aimed to develop accelerometer-based mechanical loading prediction equations for pGRF and pLR, for jumping exercises of different types and heights. We also tested different accelerometer placements and data derived from both the resultant and vertical acceleration vectors. Our results showed that the pGRF prediction models developed for jumping activities based on accelerometry data have an MAPE around 13%. The prediction equations developed for pLR resulted in a lower accuracy, with an MAPE around 22%. These models are the first to be developed specifically for high-impact jumping activities in adults.

When comparing the accuracy indices of the pGRF prediction equations developed in the present study with other equations from past investigations, developed for walking and running, it can be seen that our models have a lower accuracy [17,18,20,21,22]. This can be explained, at least partially, by the high heterogeneity in the jumping movement patterns, as several combinations of jump types and heights were tested in our study. In addition, subjects with different skill levels were included in the sample, which may have favored heterogeneity. Nevertheless, these MAPE values are similar to the results usually found in accelerometer-based energy expenditure prediction models [37], which have widespread use in research. As for the pLR prediction equations, they presented a lower accuracy compared to the pGRF equations, corroborating previous findings [19,21,22]. This is possibly due to the fact that the loading rate is typically calculated through a derivative of the force signal from the beginning of the foot’s contact with the floor to the point where the pGRF is achieved [19,21,22,38]. As this is a small time window—usually less than 0.2 s for the jumps that were performed in our protocol—it requires a fine sampling acquisition that captures small graduations in the force values throughout time, which is difficult to achieve with common accelerometer sampling frequencies, which are usually 100 Hz at maximum. Nevertheless, although our pLR prediction models had worse accuracy compared with the pGRF models, their accuracy was similar or even better than the results from previous studies that developed models to predict pLR during walking only [19,21]. Furthermore, although our prediction equations’ error was relatively low, it was accompanied by a high dispersion, as shown by its standard deviation values. This dispersion, however, is somewhat expected, given that our data included several types and heights of jumps, performed with no restriction to arm movements. Nevertheless, we have opted to include this wide range of movement patterns in order to increase the external validity of the equations, conferring them with a greater usability, without, for example, the need for different models for different kinds of jumps, which would hinder the application of the models to real-world data.

Bone increases its mass and optimizes its geometry and microarchitecture in response to adequate mechanical stimulation [1,2], particularly of high magnitude [39,40]. These types of high-intensity impact activities have been shown to be more strongly associated with bone health improvements, even if they are only performed in small volumes throughout the day. In fact, Vainionpää et al. [39] demonstrated that performing a relatively low number (<100) of impacts with pACC above 4.9 *g* daily is positively associated with increases in bone mineral density. Usually, this acceleration magnitude can be achieved only by specific movements, such as jumps [16,39]. Nevertheless, accelerometry-based mechanical loading regression models have mostly been validated for a set of activities such as walking and running, which elicit relatively low acceleration magnitudes [18,21,22]. The results of our study provide new mechanical loading prediction models for jumping, which is noteworthy because jumping is one of the most popular and effective exercises prescribed for bone health improvement [41], but also one that could increase the risk of fracture in patients with severe osteoporosis and bone fragility. Therefore, it is crucial to develop strategies that can adequately monitor the mechanical loading associated with this activity.

The development of such models was performed with the direct purpose of assessing the loading sustained by the skeletal system during daily physical activity. To date, physical activity monitoring and exercise prescription aiming for bone health promotion have largely been carried out almost blindly or through the use of subjective instruments such as questionnaires. Moreover, the absence of a trustworthy method to monitor daily mechanical loading may preclude the prescription and self-involvement of patients in high-impact activities that may expose them to a higher risk of musculoskeletal injuries. These issues may be overcome with the recent popularization of wearable sensors, such as accelerometers, and their ability to be used as a tool for mechanical loading prediction. This would allow some important clinical applications, as the models developed in this study—when used in combination with other prediction equations for other types of activities [22]—can be used to fully analyze the mechanical loading spectrum to which people are subjected daily and relate this information with bone health parameters. Therefore, a more complete understanding can be achieved as to how mechanical loading influences bone health by, for instance, identifying the loading volume and magnitude range that are more efficient in inducing bone health benefits. Moreover, the development and distribution of an R package that automates the steps necessary to apply these models enhances their applicability to potential users [16]. This can prove to be an important tool, as the application of these models depends on several laborious steps—such as the application of digital filters to reduce noise from the signal, the detection of the peaks in this signal, and the computation of derivatives to obtain the acceleration rate—necessary for the pRL prediction.

In our protocol, three accelerometer placements (ankle, lower back, and hip) and two vectors (resultant and vertical) were tested. Overall, the prediction models’ performance was reasonably consistent among the different placement and vector combinations, allowing some conclusions to be drawn. First, the hip accelerometer placement, which has traditionally been widely employed in research for the direct measurement of physical activity intensity [42], yielded a slightly better accuracy compared to the other placements tested. This is useful considering that previous data collected from hip-worn accelerometers for other purposes can be adequately reanalyzed to obtain mechanical loading predictions. Furthermore, studies that aim to extract mechanical loading together with other variables derived from accelerometry data, and that use the hip as the default accelerometer placement, can reliably use this placement. Second, as the magnitude of the resultant vectors of both pGRF and pLR in the activities that were tested is derived mostly from the vertical vector, and there were no considerable differences in performance between models using both vectors, they can be used interchangeably to predict mechanical loading. In addition, while models using data from the vertical vector allow the use of uniaxial accelerometers, data derived from the resultant vector have the important advantage of not depending on the correct orientation of the accelerometer’s axes, which cannot be guaranteed during data collection in free-living conditions [43].

Although the models presented in this study proved to be valid, some limitations ought to be considered. First, the choice of activity to elicit high-impact loading was jumping, and several combinations of jump type and height were tested. Nevertheless, the results presented here cannot be assured for other high-impact activities, or even for other types of jumps that were not tested. Second, no external sample for model validation was recruited. However, the leave-one-out cross-validation method that was used is the recommended strategy in these situations [33]. Third, to apply these models in accelerometer-recorded free-living physical activity data, there would be a need to identify the periods in the acceleration signal in which the activity being performed is jumping. Therefore, other methods for physical activity pattern detection based on accelerometer data, such as the models developed by Wang et al. [44], should be used in combination with these models to allow accurate prediction. Finally, as the jumps were always performed in the same order, the subjects may have experienced fatigue, especially in the last few trials, which could have led to changes in their jumping movement patterns.

In conclusion, accelerometry-based mechanical loading prediction models, especially for pGRF, have proven to be a valid method for determining the mechanical stimulation induced by jumps of various types and heights. These prediction models were validated using data from both the resultant and vertical vectors and with accelerometers placed at the ankle, lower back, and hip. The results from this study will enable the continuous assessment of mechanical loading in clinical settings, providing a means to objectively determine the osteogenic potential of daily physical activity and to better monitor and prescribe exercise aimed to improve bone health. Compared to pGRF, the prediction of pLR showed lower accuracy.

## Figures and Tables

**Table 1:** Regression equations, R^2^, and accuracy indices for prediction of pGRF and pLR based on accelerometer-derived data.

Vector	Accelerometer Placement	Regression Equations	R^2^	MAE	MAPE	RMSE
pGRF prediction equations						
Resultant	Ankle	pGRF (N) = 1551.020 − 132.384(pACC) + 7.927(body mass) + 2.415(pACC × body mass)	0.84	341.2 ± 275.1	13.9% ± 13.4%	438.2 ± 569.4
	Lower back	pGRF (N) = −350.125 + 152.952(pACC) + 22.618(body mass) + 0.654(pACC × body mass)	0.92	376.3 ± 257.9	14.5% ± 10.7%	456.1 ± 508.5
	Hip	pGRF (N) = −493.877 + 188.759(pACC) + 18.008(body mass) + 1.279(pACC × body mass)	0.90	302.1 ± 257.2	12.3% ± 13.4%	396.6 ± 549.2
Vertical	Ankle	pGRF (N) = 1662.525 − 196.301(pACC) + 8.515(body mass) + 3.169(pACC × body mass)	0.83	350.4 ± 282.8	14.4% ± 14.5%	450.1 ± 581.0
	Lower back	pGRF (N) = −287.919 + 131.396(pACC) + 24.338(body mass) + 0.642(pACC × body mass)	0.90	371.0 ± 257.1	14.4% ± 10.9%	451.3 ± 509.8
	Hip	pGRF (N) = −786.169 + 177.403(pACC) + 23.953(body mass) + 1.355(pACC × body mass)	0.88	322.9 ± 273.3	13.3% ± 15.0%	422.9 ± 578.1
pLR prediction equation						
Resultant	Ankle	pLR (N⋅s^−1^) = 71932.438 − 218.268(pAR) + 74.463(body mass) + 3.474(pAR × body mass)	0.88	18,973 ± 14,494	23.4% ± 26.6%	23,868 ± 29,433
	Lower back	pLR (N⋅s^−1^) = −1161.976 + 22.804(pAR) + 624.413(body mass) + 2.135(pAR × body mass)	0.89	20,320 ± 14,799	23.9% ± 23.6%	25,132 ± 28,807
	Hip	pLR (N⋅s^−1^) = 5118.300 + 33.054(pAR) + 346.667(body mass) + 2.835(pAR × body mass)	0.91	16,812 ± 13,485	20.7% ± 24.5%	21,546 ± 27,240
Vertical	Ankle	pLR (N⋅s^−1^) = 58864.225 − 194.575(pAR) + 142.545(body mass) + 3.733(pAR × body mass)	0.87	18,147 ± 14,387	23.1% ± 28.8%	23,152 ± 29,707
	Lower back	pLR (N⋅s^−1^) = 8303.550 − 19.708(pAR) + 685.299(body mass) + 1.900(pAR × body mass)	0.88	21,001 ± 14,831	24.7% ± 25.2%	25,704 ± 28,869
	Hip	pLR (N⋅s^−1^) = −11471.926 + 15.332(pAR) + 691.269(body mass) + 2.670(pAR × body mass)	0.88	18,801 ± 15,478	22.9% ± 27.5%	24,345 ± 30,582

Abbreviations: MAE, mean absolute error; MAPE, mean absolute percentage error; pACC, peak acceleration; pAR, peak acceleration rate; pGRF, peak ground reaction force; pLR, peak loading rate; RMSE, root-mean-square error. Data: mean ± standard deviation.

## Data Availability

The data presented in this study are available on request from the corresponding author.

## Supplementary Materials

The following supporting information can be downloaded at: https://www.mdpi.com/article/10.3390/s23042246/s1, Figure S1: Distribution of the vertical vector of mechanical loading (peak ground reaction force on Panel A and peak loading rate on Panel B) and acceleration (peak acceleration on Panel C and peak acceleration rate on Panel D) variables per jump type and height combination. Abbreviations: BW, body weight; pACC, peak acceleration; pAR, peak acceleration rate; pGRF, peak ground reaction force; pLR, peak loading rate; Figure S2: Bland-Altman plots showing agreement between actual and predicted peak ground reaction force for accelerometers worn at ankle (Panels A and D), lower back (Panels B and E) and hip (Panels C and F), for both the resultant vector (Panels A to C) and its vertical component (Panels D to F). Continuous thick lines show bias (average of the differences between actual and predicted values) while dashed lines show the limits of agreement (bias ± 1.96 standard deviation). Abbreviations: pGRF, peak ground reaction force; Figure S3: Bland-Altman plots showing agreement between actual and predicted peak loading rate for accelerometers worn at ankle (Panels A and D), lower back (Panels B and E) and hip (Panels C and F), for both the resultant vector (Panels A to C) and its vertical component (Panels D to F). Continuous thick lines show bias (average of the differences between actual and predicted values) while dashed lines show the limits of agreement (bias ± 1.96 standard deviation). Abbreviations: pLR, peak loading rate.
