# Supplementary material for: Using Raw Accelerometer Data to Predict High-Impact Mechanical Loading
Source: Sensors (Basel). 2023 Feb 16;23(4):2246. doi: 10.3390/s23042246 (PMC9960291; doi:10.3390/s23042246)
Supplement: Supplementary file 1 [file sensors-23-02246-s001.zip › sensors-2036813-supplementary.pdf]

Supplemental Material

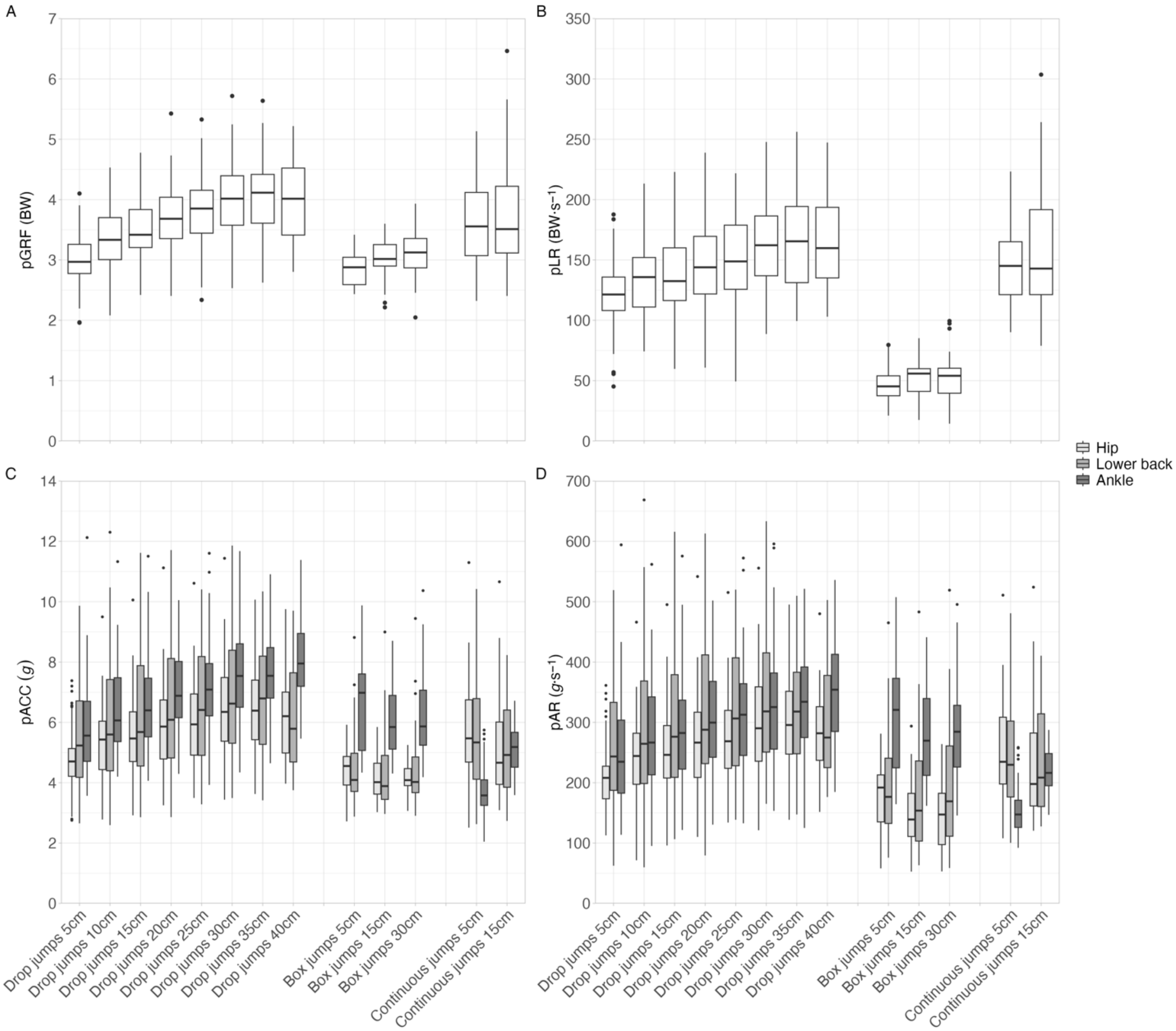

Supplemental Figure S1. Distribution of the vertical vector of mechanical loading (peak ground reaction force on Panel A and peak loading rate on Panel B) and acceleration (peak acceleration on Panel C and peak acceleration rate on Panel D) variables per jump type and height combination. Abbreviations: BW, body weight; pACC, peak acceleration; pAR, peak acceleration rate; pGRF, peak ground reaction force; pLR, peak loading rate.

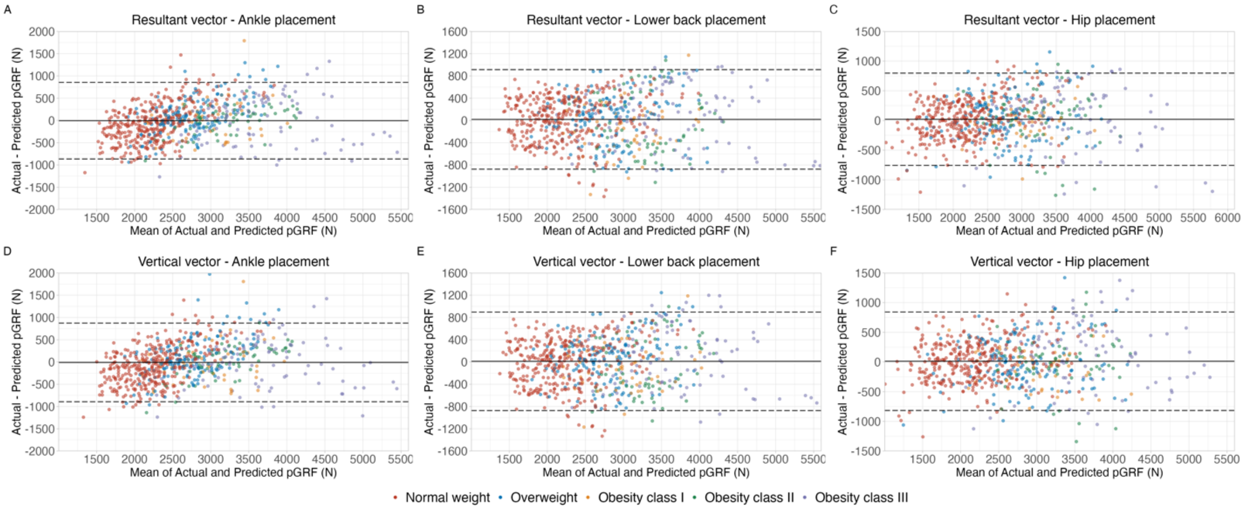

Supplemental Figure S2. Bland-Altman plots showing agreement between actual and predicted peak ground reaction force for accelerometers worn at ankle (Panels A and D), lower back (Panels B and E) and hip (Panels C and F), for both the resultant vector (Panels A to C) and its vertical component (Panels D to F). Continuous thick lines show bias (average of the differences between actual and predicted values) while dashed lines show the limits of agreement (bias ± 1.96 standard deviation). Abbreviations: pGRF, peak ground reaction force.

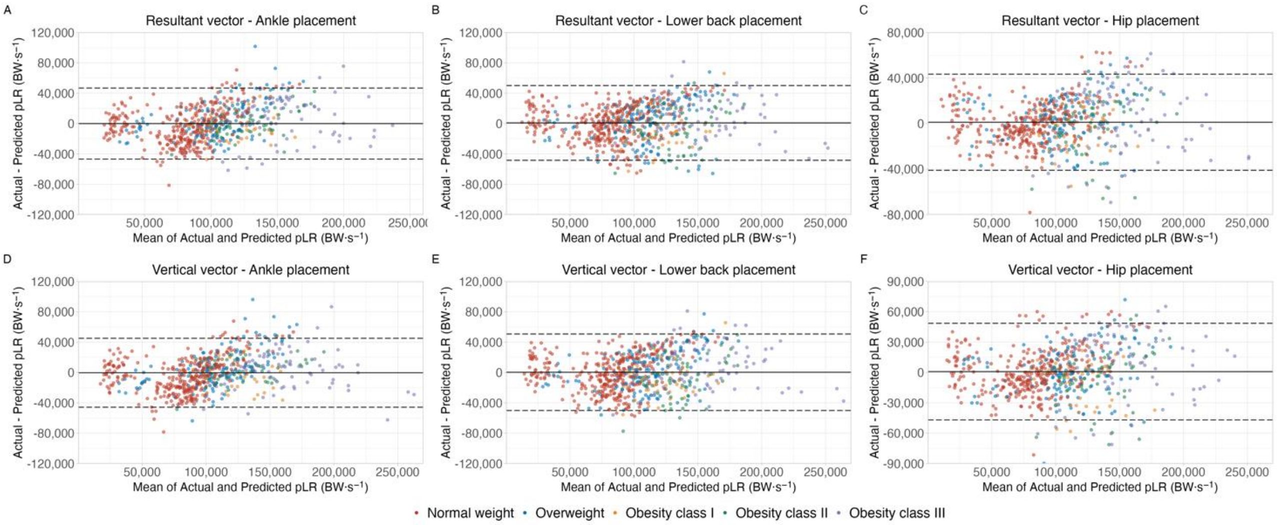

Supplemental Figure S3. Bland-Altman plots showing agreement between actual and predicted peak loading rate for accelerometers worn at ankle (Panels A and D), lower back (Panels B and E) and hip (Panels C and F), for both the resultant vector (Panels A to C) and its vertical component (Panels D to F). Continuous thick lines show bias (average of the differences between actual and predicted values) while dashed lines show the limits of agreement (bias ± 1.96 standard deviation). Abbreviations: pLR, peak loading rate.
